# Comparison of Near Infrared Spectra of Three Lichen Substances and Several Common Synthetic Organic Sunscreens

**DOI:** 10.1111/jocd.70288

**Published:** 2025-07-02

**Authors:** David Gilberto Torres Vargas

**Affiliations:** ^1^ Chemist and Biologist Universidad Nacional de Colombia Sede Bogotá DC Colombia; ^2^ Facultad de Ciencias—Departamento de Química Universidad Nacional de Colombia Sede Bogotá DC Colombia

**Keywords:** claim, lichen substances, *Lobariella sipmanii*, near‐infrared spectroscopy, structure, sunscreens

## Abstract

**Background:**

Near‐infrared (NIR) and UV radiation have been reported to alter skin physiology, causing oxidative stress, mitochondrial genetic damage, and potentially leading to cancer and photoageing. NIR screening is recommended, but only inorganic sunscreens such as zinc and titanium oxide are known to absorb, reflect, and scatter NIR. Additionally, NIR spectra of common organic sunscreens are neither available in the literature nor in technical datasheets.

**Aim:**

This study characterizes NIR absorption of several commercial sunscreens and alternative lichen compounds.

**Methods:**

To measure NIR absorbance and reflectance spectra, solid organic sunscreens were impregnated with minimal chloroform onto pure dry potassium bromide (KBr) crystals and analyzed using a Varian Cary 5000 spectrophotometer. Liquid compounds were tested directly in standard 1 cm quartz cells. Reflectance spectra were processed using the Kubelka‐Monk equation.

**Results:**

Sunscreens and lichen compounds effectively absorb radiation beyond 1500 nm, especially above 2100 nm, with less‐substituted aromatic compounds such as sunscreens and methyl orsellinate performing better than more substituted ones like pseudocypherellin A and methyl 2,4‐dihydroxy‐3,5,6‐trimethylbenzoate. Additionally, less‐substituted compounds exhibited minor absorption bands between 1000 and 1500 nm.

**Conclusions:**

Compounds with hydroxyl, carbonyl groups, medium‐length aliphatic chains, and low degree of aromatic substitution may provide NIR photoprotection. Owing to their antioxidant properties, lichen compounds are better alternatives. NIR spectroscopy is also suitable for quantification and quality control of sunscreens due to their high concentrations in finished products. However, there is still a need to develop or discover new organic sunscreens with strong NIR absorption.

## 1 Introduction

Solar radiation spans several wavelengths of the electromagnetic spectrum, and its primary target on the human body is the skin [1]. Exposure to ultraviolet (UV‐R) and infrared radiation (IR) can induce DNA damage, oxidative stress, and inflammatory processes that ultimately lead to skin pathologies [1, 2, 3, 4, 5]. The biological effects of IR resemble the biological endpoints of UV irradiation [5]; moreover, chronic exposure to IR increases skin temperature sufficiently to cause skin problems, such as thermal keratosis, erythema *ab igne*, extracellular matrix degradation, photoageing, and basal cell carcinoma, among others [6, 7, 8, 9, 10, 11]. Most IR exposure effects are caused by oxidative stress due to disruption of the mitochondrial respiratory chain and changes in fibroblast gene expression, which modify metabolism of the extracellular matrix, causing failure of elastic fibers and collagen synthesis, induction of inflammatory cytokines, irregular pigmentation, and angiogenesis [1, 2, 6, 8, 9].

A common protection strategy involves the use of creams and other cosmetic products containing sunscreens that absorb, scatter, or reflect sunrays [12, 13]. Most formulations are oil‐in‐water (O/W) emulsions, such as creams, sprays, or lotions, and contain a mixture of physical and chemical sunscreens in amounts ranging from 1% to 20% wt., depending on the ingredients and current regulations [14, 15, 16]. Physical or inorganic sunscreens are oxides (e.g., titanium dioxide or zinc oxide) that reflect or scatter solar radiation, including IR [5, 10, 14]. Whereas chemical sunscreens are organic compounds that can absorb certain wavelengths of UV‐R but have limited filtering capacity, especially against shortwave IR [5, 10, 14].

Near‐infrared radiation (NIR) accounts for approximately 20%–50% of incident sunrays and encompasses light between 700 and 3000 nm (14 000 cm^−1^–4000 cm^−1^). Although NIR has lower energy (6.6 × 10^−20^–1.9 × 10^−19^ J/photon) than UV‐R (5.6–6.6 × 10^−19^ J/photon) [17], it receives little attention in photoprotection despite its harmful effects, and its underlying mechanisms have not been as extensively studied as for UV‐R [1, 5, 18, 19]. Moreover, NIR, visible light, and UV‐A are not absorbed nor dispersed by the keratin of *stratum corneum*, reaching deep skin layers [1, 4, 20]. Indeed, the dermis absorbs most of the shorter IR (760–1400 nm) [1, 2, 17, 21].

While most chemical sunscreens absorb UV‐B radiation (280–320 nm), dibenzoylmethanes (e.g., Avobenzone—AVO) are the preferred option for absorbing UV‐A radiation (320–400 nm) [22], and broad‐spectrum sunscreens (e.g., Bemotrizinol—BMT) cover almost the entire UV range (280–400 nm) [14, 22]. However, only a few compounds can absorb in the shortest wavelength of the NIR or NIR region I (700–1000 nm); therefore, this radiation is highly transmitted [23].

Organic compounds absorb NIR mainly from overtones and combination bands from the vibrational transitions of hydrogen‐containing groups (C‐H, O‐H, and N‐H), which can be explained by the non‐ideal nature of the chemical bonds and the resulting anharmonicity in vibrational transitions [18, 19, 23, 24, 25, 26]. Some electronic transitions also occur in this region and are typically derived from π‐π* transitions in highly conjugated systems or charge‐transfer transitions. Despite the widespread use of chemical sunscreens, their NIR absorption spectra are not available and remain unknown [5, 27]; therefore, their NIR screening ability has not been determined.

Current organic sunscreens present adverse effects such as toxicity, allergenicity, photosensitisation, immunosuppression, and environmental concerns, prompting interest in natural products as alternatives to conventional sunscreens [28, 29, 30, 31, 32]. Lichenized fungi such as *Evernia prunastri* and *Pseudoevernia furfuracea* have been extensively used in the perfume industry [33]. Lichens thrive in environments with elevated solar radiation, such as high mountains, tundra, polar, and coastal regions; and biosynthesise considerable amounts of phenolic compounds, up to 30% wt. dry thalli [34, 35, 36]. Thus, lichen substances could be particularly promising as sunscreen alternatives. Hence, this study aims to obtain NIR spectra to evaluate whether currently available sunscreens and lichen alternative substances can effectively absorb NIR.

## 2 Methods

NIR spectra were recorded using a Varian Cary 5000 spectrophotometer (Agilent Technologies) with wavelength increments (resolution) of 1 nm in transmittance mode for octyl methoxycinnamate and in reflectance mode for solid samples. To minimize interference from carbon‐hydrogen and hydrogen bonds present in common organic solvents, compounds can be dissolved in polychlorinated or deuterated solvents to record NIR spectra [18, 23, 26]. Humidity must also be avoided owing to the strong molecular absorptivity of water in the NIR region and its influence on hydrogen bonding in other molecules [23, 37].

Liquids can be measured directly or dissolved in the aforementioned solvents at moderate concentrations in quartz standard spectrophotometric cells [18, 23, 26]. The transmittance spectrum of the liquid compound (octyl methoxycinnamate—OMC‐EHMC) was taken directly in a 1 cm quartz spectrophotometric cell and then converted into absorbance using Lambert–Beer law (Equation 1).

(1) $$A=2-\log_{10}T$$

For small amounts of solid compounds, solid preparations (powders, pellets, or tablets) in potassium bromide or any IR‐transparent substrate can be used. Each solid preparation was made from 10 mg of pure compound dissolved in a minimal amount of chloroform, which was used to impregnate approximately 100 mg of pure dry potassium bromide (KBr) crystals (Merck‐Millipore). The impregnated crystals were then heated in a drying oven at 40°C for 1 h. The resulting powders were stored in a desiccator and then measured directly in a powder spectrophotometric cell.

The lower molar absorptivity in IR than in UV/visible range has two consequences: First, the product of molar absorptivity and concentration is small, limiting IR spectroscopy to concentrated samples. Second, this also allows the validity of equations relating solid diffuse reflectance to absorbance, such as the Kubelka‐Munk equation (Equation 2) [24]. The Kubelka‐Munk equation was used to calculate powder absorbance from the recorded reflectance data of solid compounds [24, 38].

(2) $$f\left(R\right)=\frac{\left(1-R^{2}\right)}{2R}$$

Nonetheless, the crystals of pure potassium bromide have their own effect on reflectance. Therefore, a baseline measurement with pure dry potassium bromide was performed to correct the spectra of solid compounds, and the Kubelka‐Monk equation was also applied (Equation 3).

(3) $$f{\left(R\right)}_{\text{corrected}}=f{\left(R\right)}_{\text{sample}}-f{\left(R\right)}_{\text{Pure}\;\mathrm{KBr}}$$

Blanco et al. suggested that apparent absorbance can be calculated directly from reflectance data using a concentration/reflectance relationship similar to the Lambert–Beer law, which was also evaluated in this study (Equation 4) [24].

(4) $$\text{Aparent Absorbance}=\log\;\frac{1}{R}=a^{\prime }C$$

**FIGURE 1:**
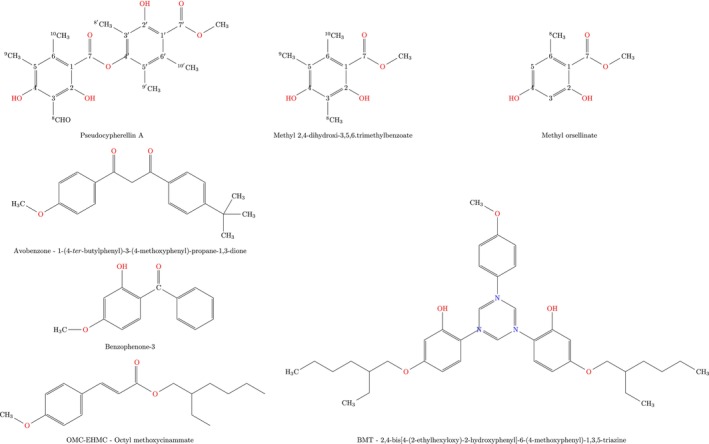
Chemical structures of the studied substances. For *Lobariella sipmanii* compounds, the corresponding nomenclature of carbon atoms is given.

Lichen substances were isolated from 2.8 g of acetonic extract of *Lobariella sipmanii* (Moncada, Betancourt & Lücking) using an open silica gel column. The structures of the compounds were confirmed by ^1^H‐ and ^13^C‐NMR spectra, and their purity was assessed through TLC and melting point analysis. The lichen material was collected in Paramo de Sumapaz (Southern Bogota DC—Colombia) at an altitude of 3800 m above sea level (COL: 609185). Commercial sunscreens: Octyl methoxycinnamate—OMC‐EHMC (CAS Number: 226–775‐7), bemotrizinol—BMT (CAS Number: 187393–00‐6), avobenzone—AVO (CAS Number: 274–581‐6), and benzophenone‐3—BP3 (CAS Number: 131–57‐7) were supplied by their respective manufacturers (Figure 1).

### 2.1 Isolated Compounds

The NMR spectra of the *Lobariella sipmanii* compounds are presented, and their nomenclature is defined in Figure 1.

**Pseudocypherellin A:** ^**1**^ **H‐NMR (CDCl**_**3**_ **–400 MHz) δ:** 2.09 (3H, s, CH_3_‐9′); 2.11 (3H, s, CH_3_‐10′); 2.21 (3H, s, CH_3_‐9); 2.50 (3H, s, CH_3_‐8′); 2.73 (3H, s, CH_3_‐10); 4.01 (3H, s, COOCH_3_); 10.40 (1H, s, CHO); 11.14 (1H, s, OH‐2′); 12.41 (1H, s, OH‐4); 13.10 (1H, s, OH‐2). ^**13**^ **C‐NMR (CDCl**_**3**_ **) δ:** C‐1: 102.8; C‐2: 166.9; C‐3: 107.9; C‐4: 166.1; C‐5: 118.2; C‐6: 150.1; C‐7: 169.8; C‐8: 194.0; C‐9: 10.8; C‐10: 20.5; C‐1′: 111.9; C‐2′: 158.9; C‐3′: 116.2; C‐4′: 151.5; C‐5′: 120.5; C‐6′: 137.6; C‐7′: 172.1; C‐8′: 18.9; C‐9′: 13.3; C‐10′: 9.8; C7′‐COOMe: 52.3. **Melting point:** 174°C.

**Methyl 2,4‐dihydroxy‐3,5,6‐trimethyl benzoate (2,4‐DH):** ^**1**^ **H‐NMR (CDCl**_**3**_ **–400 MHz) δ:** 2.13 y 2.14 (6H, ds, CH_3_‐8, CH_3_‐9); 2.43 (3H, s, CH_3_‐10); 3.92 (3H, s, COOCH_3_); 11.46 (1H, s, OH‐2). ^**13**^ **C‐NMR (CDCl**_**3**_ **) δ:** C‐1: 106.3; C‐2: 159.7; C‐3: 107.4; C‐4: 156.8; C‐5: 115.0; C‐6: 137.7; C‐7: 172.9; C‐8; 12.0; C‐9; 8.1; C‐10: 18.9; C7‐COOMe: 52.0. **Melting point:** 138°C.

**Methyl orsellinate:** ^**1**^ **H‐NMR (d6‐acetone—400 MHz) δ:** 2.47 (3H, s, CH_3_‐8); 3.93 (3H, s, COOCH_3_); 6.25 (1H, s, C3); 6.30 (1H, s, C5); 9.12 (1H, s, OH‐4); 11.64 (1H, s, OH‐2). ^**13**^ **C‐NMR (d6‐acetone) δ:** C‐1: 105.4; C‐2: 166.2; C‐3: 101.3; C‐4: 163.4; C‐5: 112.3; C‐6: 144.3; C‐7: 173.1; C‐8: 24.0; C7‐COOMe: 52.0. **Melting point:** 97°C.

## 3 Results and Discussion

**FIGURE 2:**
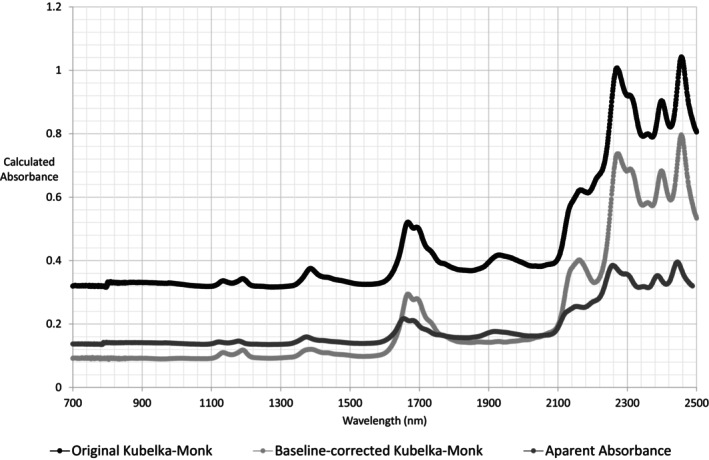
Effects of different NIR spectral processing methods for AVO.

In terms of spectral processing, the Kubelka‐Monk equation is the most advisable method for determining absorbance; however, a baseline correction must be applied to remove the potassium bromide bands, as shown in Figure 2. This correction is also necessary because of the particle size influence on the baseline, where larger particles present stronger absorption, and which is relevant for quantitative analysis [39]. The Kubelka‐Monk equation can be applied to water and other analytes with high NIR molar absorptivity (e.g., organic compounds) and has advantages such as a response independent of particle shape and linearity at different concentrations [38]; this latter aspect should be further investigated for sunscreens. Conversely, the apparent absorbance method diminishes the calculated absorbance, especially for intense bands, and the resulting spectrum lacks several details and overtones, despite the apparent absorbance being mathematically simpler than the Kubelka‐Monk equation.

**FIGURE 3:**
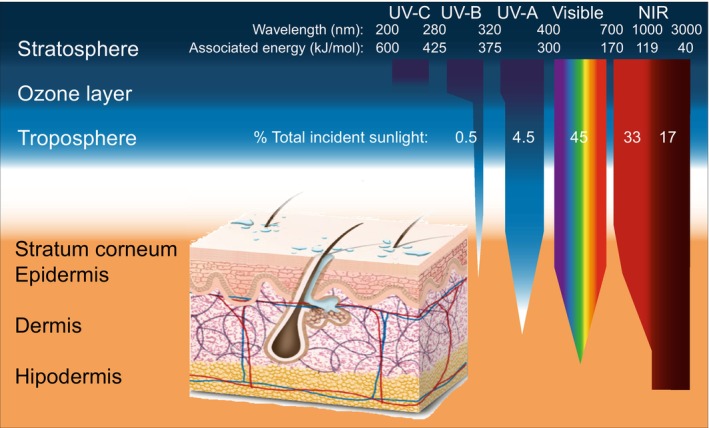
Atmosphere and skin absorption of different wavelengths of sun radiation and their associated energies in kJ mol^−1^. Wavelength and energy values were adapted from Schroeder & Krutmann, 2010; Sklar, 2013; and Stiefel & Schwack, 2015 [5, 7, 10].

Despite the well‐known physiological effects of NIR and its association with photoageing and some types of skin cancer [1, 2, 7, 17, 21, 40]. NIR spectra of organic UV sunscreens are scarce in the literature and technical datasheets [27]. This study presents the absorbance and reflectance spectra of four common sunscreens and three natural alternatives from *Lobarella sipmanii*, a lichenized fungus from Paramo. Shortwave NIR or infrared radiation between 700 and 1000 nm, which represents about one third of total sun rays, can penetrate the skin and reach the subcutaneous tissue; in fact, at least half of shortwave NIR is absorbed in the dermis (Figure 3) [2, 5, 13, 21].

**FIGURE 4:**
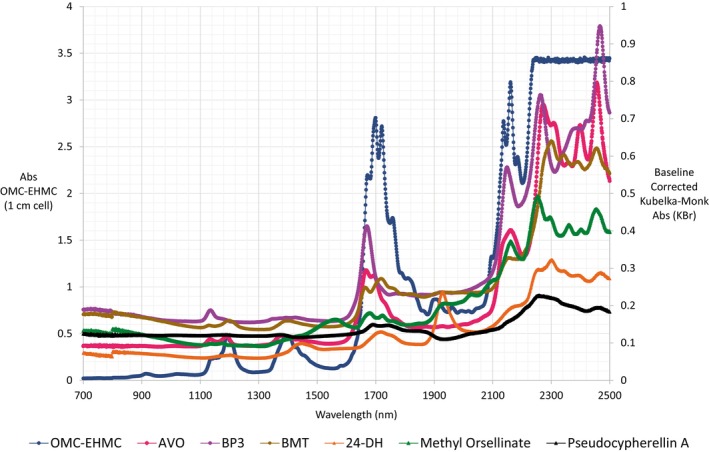
NIR absorbance spectra. Solid compounds have their absorbance‐axis on the right side. Octyl methoxycinnamate (Blue) was the only liquid compound, and its absorbance‐axis is on the left side.

All organic sunscreens and *Lobariella sipmanii* compounds evaluated showed low NIR absorption in this region (Figure 4), which is consistent with the reported transparency of most organic compounds in NIR Region I [23]. Consequently, the compounds evaluated did not protect NIR Region I skin chromophores, such as mitochondrial cytochrome oxidase or copper atoms in complex IV of the respiratory chain [5, 27]. NIR irradiation of the mitochondrial electron transport chain generates reactive oxygen species (ROS) and leads to oxidative stress [2, 17, 41, 42, 43].

Systems with multiple NH or OH groups have separate NIR absorption bands that are readily distinguishable from the mid‐IR bands (> 2500 nm) [23]. Phenolic hydroxyls present first overtone bands at 1400–1440 nm and combination bands near 2000 nm [18, 19]. Phenolic compounds such as *Lobariella sipmanii* compounds (2,4‐DH and methyl orsellinate) and BMT have these discrete, broad absorption bands corresponding to first overtones, as shown in Figure 4. Monoaromatic compounds have these bands at 1430 nm (2,4‐DH) and at 1550 nm (methyl orsellinate). Rounded combination bands appear at 1920 nm for 2,4‐DH, whereas methyl orsellinate has a band at 2144 nm before the region of higher NIR absorption. The second overtones are less intense and probable, appearing around 1000 nm [19]; however, they were not observed in our spectra. Hydrogen bonding influences the intensity and broadness of these bands; compounds with stronger association result in broader, less intense bands [23, 26].

C‐H stretching overtones allow the prediction of various C‐H bond classes, distinguishing between methyl, methylene, methine, and aromatic groups [18, 26]. The first overtones occur at 1700–1800 nm, the second overtones occur at 1200 nm, and the third overtones appear between 880 and 915 nm, which are stronger in longer hydrocarbon chains [19]. Compounds with larger hydrocarbon chains, OMC, and BMT exhibited pronounced first overtone signals, with visible second overtones (Figure 4). Shorter hydrocarbons (AVO, OMC‐EHMC and also BMT) displayed two second overtone bands of similar size, one corresponding to the asymmetric stretching overtone of methylene or methine and the other belonging to methyl groups [19]. Alkyl aromatic substitution leads to mixed aromatic‐aliphatic characteristics in the spectra [19]. This is evident for all compounds; however, the more substituted compounds have predominantly aliphatic features in their spectra.

The second NIR region includes the overtones of the mid‐IR stretching vibrations and the combination modes, while region III encompasses combination modes and the first and second stretching overtones of C=O or C≡N groups [19, 23]. The aromatic compounds have overtones at 1685 and 1143 nm and combination bands at 1420–1450, 2150, 2460 nm [18, 19]. In assayed commercial sunscreens, aromatic overtones were observed around 1150–1190 nm and 1660–1726 nm. All compounds had overtones above 2150 nm. Aromatic compounds with less than four substituents, such as methyl orsellinate and commercial sunscreens, exhibited a small absorption band between 1000 and 1500 nm in NIR region II. Nevertheless, *Lobariella* substances 2,4‐DH and pseudocypherellin A only presented the overtone between 1660 and 1800 nm; highly substituted aromatic rings absorbed IR in this region to a lesser extent than less‐substituted rings (Figure 4). This suggests a hypochromic effect in NIR, similar to that found in UV for these lichen substances (Data not shown), due to their higher methyl substitution.

Carbonyl and cyano groups present intense overtones due to their dipole moments, with carbonyl overtones at 1900–1960 nm [19, 23]. Sunscreens with carbonyl groups as ketones or esters, such as AVO, BP3, or OMC‐EHMC, show higher NIR absorption than sunscreens without carbonyls, like BMT. All the compounds studied had intense bands in the long‐wavelength NIR region or region III (> 1500 nm), particularly above 2100 nm (Figure 4). Electron‐donating or withdrawing groups cause bathochromic or hypsochromic effects in the NIR, respectively [19]. Polymethylated compounds such as pseudocypherellin A and 2,4‐DH had overtones between 1720 and 1760 nm, while methyl orsellinate and commercial sunscreens with electron‐withdrawing (carbonyl) groups absorbed radiation near 1650–1700 nm.

The aforementioned bands below 1500 nm may be relevant for shortwave NIR photoprotection. Shortwave IR regulates the expression of at least 21 extracellular matrix homeostatic proteins, including fibronectin, VCAM‐1, and cadherins, which exhibit reduced expression after IR‐A exposure [1]. Matrix metalloproteinases such as MMP‐1, MMP‐2, and MMP‐12 and their inhibitory proteins (TIMPs) are also altered in their expression, reducing the production of new collagen fibers via retrograde signaling and contributing to the expected photoaged skin changes [1, 6, 9, 17, 40, 41, 43]. While elastic components are over‐expressed, leading to the accumulation of non‐functional elastic fibers in photoaged skin [5, 6]. Therefore, effective IR‐A screening can prevent ROS production, matrix metalloprotease upregulation, IR‐induced inflammatory response, angiogenic switch, and eventual photoageing [2, 13, 27].

Despite the current claims of NIR photoprotection in many commercial sunscreens, the role of NIR in skin cancer remains controversial [27]. For instance, prior irradiation with IR exposure reduces UV‐R‐induced oxidative stress and activates skin protective systems [6, 8, 21, 27]. However, Jantschitsch et al. found that IR‐A treatment prior to UV‐R exposure reduced apoptosis in murine keratinocytes [21], and NIR augmented the expression of antiapoptotic proteins like TNFRSF6B, potentially attenuating cell death after IR and UV exposure, and contributing to photocarcinogenesis [1, 21, 27].

ROS production is the dominant process after sun exposure across the entire spectrum [2, 3, 7, 8, 27]. IR‐A is strongly absorbed by mitochondria and induces damage by disrupting the respiratory electron transfer chain, leading to inadequate energy production, excessive ROS generation, and eventual retrograde signaling [2, 6, 8, 27, 41]. Damaged respiratory proteins further accelerate ROS production and mitochondrial genome mutations, particularly large‐scale deletions, which are strongly associated with sunlight exposure rather than normal chronological aging [41]. Additionally, mitochondria lack the repair mechanisms required to remove large‐scale mtDNA damage, causing cumulative damage that alters their gene expression and function, creating a vicious cycle of increased ROS production and further mitochondrial damage, ultimately leading to cell dysfunction [41]. ROS are also second messengers in cellular signaling pathways, and they inactivate some intracellular phosphatases [5].

Although shortwave NIR (IR‐A) is the predominant cause of IR‐induced ROS production and retrograde signaling, other IR wavelengths and UV‐R also contribute [27]. In addition, NIR depletes skin antioxidants, like carotenoids, but thermal destruction of these substances does not occur [3, 8]. Hence, antioxidant activity is crucial to prevent mitochondrial ROS generation and the consequent changes in cell signaling and gene expression. Schroeder et al. suggested that topical application of antioxidants, especially polyphenols, mitigates the changes in MMP‐1 expression [5, 40]. Hence, antioxidant activity complements NIR absorption and is a desirable feature of plant extracts and sunscreen alternatives [1, 13, 29, 41].

Most current organic sunscreens lack antioxidant properties, and some may act as pro‐oxidants, undergo photo‐oxidation, or photo‐isomerisation reactions, or be photo‐unstable [29, 44, 45, 46, 47]. Some also raise safety concerns such as allergenicity, endocrine disruption, and bioaccumulation [28, 30, 32, 48, 49]. Conversely, phenolic compounds are recognized antioxidants; 2,4‐DH and pseudocypherellin A showed moderate ferric ion reducing power (FRAP), moderate scavenging activity in the DPPH assay, and lipid peroxidation inhibition comparable to that of tocopherol phosphate (Vitamin E) and BHT at 24 h (data not shown). Thus, they could be considered for NIR photoprotection owing to their moderate NIR absorption and antioxidant activity, which could prevent IR‐induced oxidative stress as well as kinase‐mediated signaling changes [5]. Methyl orsellinate may also be suitable for NIR protection, although it lacks antioxidant properties [50, 51]. Lichen compounds, such as atranorin and usnic acid, are already used in the cosmetic and fragrance industry, but atranorin may cause contact allergy [49]. Nevertheless, specific structural features, such as electrophilic groups, require careful attention in safety assessment, and the allergenic potential of new lichen compounds remains to be investigated [28, 52].

**FIGURE 5:**
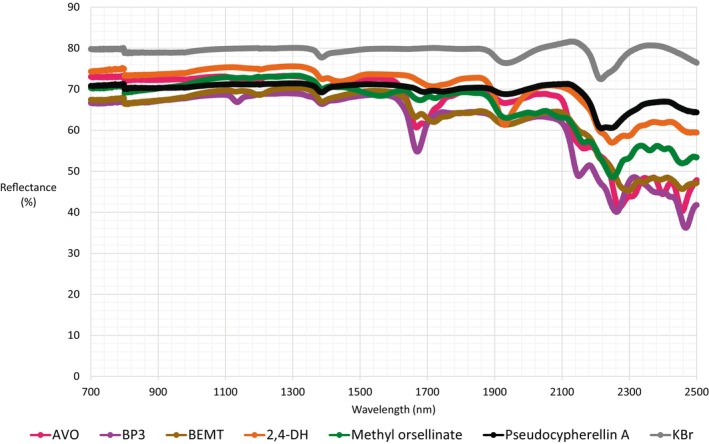
NIR reflectance spectra of solid compounds impregnated in KBr crystals.

The reflectance spectra must include the baseline because the impregnated potassium bromide powder was used (Figure 5). The differences in reflectance between pure potassium bromide and the samples were attributed to the sum of sample absorbance and transmittance. Considering the potassium bromide concentration (about 90%), we assumed that the transmittances of pure potassium bromide and the samples are almost the same, and the differences may be attributed to the absorbance of sunscreens and lichen compounds. Up to 1700 nm, all the solid compounds evaluated showed similar reflectance behavior; however, the reflectance decreased to a greater extent for those compounds with strong NIR absorptions, as was observed for the carbonyl, aliphatic, and aromatic group absorption bands compared to the baseline.

Pseudocypherellin A with lower absorbance also exhibited larger reflectance values, indicating that IR reflectance may play a role in photoprotection for polysubstituted lichen substances, such as Pseudocypherellin A and 2,4‐DH. Nonetheless, simultaneous measurement of reflectance with transmittance or absorbance is required to confirm this hypothesis.

O/W formulations predominate in sun care products, from moisturizers to sunscreens [15]. NIR can detect water content in formulations and skin due to its distinctive absorption bands at 970, 1200, 1450 nm, and a combination band at 1940 nm [23, 53]. It has also been used to non‐invasively assess the hydration degree of the *stratum corneum* [53]. Nevertheless, water can interfere with NIR analysis of powdered preparations due to hydrogen bonding within the samples, making the hydroxyl overtone bands broader and less intense [23, 26, 37]. Consequently, water and moisture interferences must be avoided when analyzing and quantifying raw materials and pure organic sunscreens.

NIR spectroscopy offers a quick, non‐destructive, and sensitive method with minimal or no sample preparation (direct application) [23]. Processing of reflectance spectra using the Kubelka‐Monk equation is straightforward and accurate and can provide linearity from the reflectance data [24, 38], as shown above. However, the quantification limitations of NIR spectroscopy should be known, especially for concentrations below 1% [23, 24]. NIR is now being used in the pharmaceutical and cosmetic industries for raw material analysis and identification, quality monitoring, and control during production and in finished products. It is well suited to substantiate NIR absorption claims for organic sunscreens, which are typically formulated at concentrations between 1.0% and 25% [16].

## 4 Conclusions

An ideal NIR sunscreen should have low‐molecular weight, aromatic rings, short aliphatic chains (2–8 carbon atoms), and strong dipole groups, such as carbonyl or hydroxyl. Highly electrophilic groups should be avoided due to toxicity, photoinstability, and allergy risks. Minimizing aromatic substitution enhances photoprotection, whilst phenolic groups are desirable for their antioxidant and NIR absorption (1000 nm) properties, making antioxidant activity a valuable feature in NIR sunscreens.

Although *Lobariella* polyphenols such as pseudocypherellin A or methyl 2,4‐dihydroxy‐3,5,6‐trimethylbenzoate (2,4‐DH) lack some of the desirable structural characteristics for absorption, their antioxidant activity could mitigate NIR‐induced oxidative stress and cell signaling alterations; this is the most recommended approach for NIR photoprotection. Within the *Lobariella* compounds evaluated, methyl orsellinate showed the best NIR absorption, sharing several structural features with the proposed ideal NIR sunscreen but lacking antioxidant activity.

In this study, the NIR absorbance and reflectance spectra of four common UV sunscreens were recorded, which are novel to the literature. These sunscreens exhibited little absorption in the shortwave NIR (< 1100 nm); however, those with aromatic, carbonyl, and hydroxy groups exhibited strong absorption bands in the NIR III region (> 1500 nm). Sunscreens with medium‐length aliphatic chains showed second overtones at 1200 nm in the NIR spectra. Moreover, differences in reflectance could be attributed to compound absorbance; those with higher absorption have lower reflectance, but their effect should be further investigated.

Shortwave IR (NIR region I; 700–1000 nm) remains a challenge for cosmetic chemists due to the limited absorption of organic compounds. None of the organic sunscreens and *Lobariella* compounds were absorbed in this region, emphasizing the need for new substances with strong NIR overtones. Formulation excipients and their ability to form hydrogen bonds with sunscreens can also affect NIR absorption by broadening or weakening bands.

NIR spectroscopy offers a straightforward, fast, inexpensive, and non‐destructive method to evaluate the IR photoprotective ability of potential sunscreens with minimal sample preparation. This technique not only assesses sunscreen efficacy but also monitors cosmetic product quality by quantifying highly concentrated raw materials in finished products, such as organic sunscreens. Sustainable alternatives, such as deuterated solvents, can replace polychlorinated solvents for crystal impregnation.

## Disclosure

*Statement of Contribution*: The contents of the present paper were developed with original spectroscopical data, the cited references were revised and cited properly, and all procedures were performed as reported.

## Ethics Statement

The author has nothing to report.

## Conflicts of Interest

The author declares no conflicts of interest.

## Data Availability

Research data are not shared.
